# Association of *PPARγ2* Pro12Ala polymorphism with gestational diabetes mellitus risk: A systematic review and meta-analysis

**DOI:** 10.17305/bb.2025.13079

**Published:** 2025-11-18

**Authors:** Yuanting Xu, Yi Du, Tengfei Shan, Qingwen Xie, Hongli Zhu

**Affiliations:** 1 Department of Obstetrics and Gynecology, Hangzhou Linping District First People’s Hospital, Hangzhou, China; 2 Department of Obstetrics and Gynecology, Hangzhou First People’s Hospital, Hangzhou, China

**Keywords:** Gestational diabetes mellitus, *PPAR*γ*2*, Pro12Ala polymorphism, genetic susceptibility, updated meta-analysis

## Abstract

Gestational diabetes mellitus (GDM) is a prevalent pregnancy complication that poses significant risks to both mothers and their offspring, with genetic susceptibility believed to play a role in its pathogenesis. This study examined the association between the Pro12Ala (Pro [C]→Ala [G]) polymorphism in the peroxisome proliferator-activated receptor *γ2* (*PPARγ2*) gene and the risk of developing GDM. A systematic literature search was conducted across databases including PubMed/Medline, Web of Science, Embase, and the Cochrane Library, identifying clinical studies that evaluated the relationship between the *PPARγ2* Pro12Ala variant and GDM. Strict inclusion criteria ensured that all case groups comprised exclusively women diagnosed with GDM. Data on study characteristics, sample sizes, and allele frequencies were extracted, and meta-analyses were performed using RevMan 5.3 and Stata with Hartung–Knapp random-effects models. Fifteen studies were included in the analysis. The Pro12Ala polymorphism showed no significant association with GDM risk across allelic (Ala [G] vs Pro [C]), dominant (CG+GG vs CC), and recessive (GG vs CG+CC) models (allelic: OR = 0.90, 95% CI = 0.75–1.08, *P* ═ 0.26; dominant: OR = 0.92, 95% CI = 0.74–1.13, *P* ═ 0.42; recessive: OR = 0.82, 95% CI = 0.54–1.25, *P* ═ 0.33; all *P* > 0.05). Subgroup analyses by ethnicity indicated a potential protective association of the Ala (G) allele with GDM in East Asian populations, while no significant associations were found in European or Middle Eastern populations; ethnicity was identified as a significant effect modifier (*P* < 0.05). There were no meaningful differences in subgroups categorized by study quality and sample size. Sensitivity analyses confirmed the robustness of the findings, and small-study effects detected by Egger’s test did not substantially alter the pooled estimates. In conclusion, the *PPARγ2* Pro12Ala polymorphism was not significantly associated with GDM risk in the general population. The potentially protective trend observed in East Asian women warrants cautious interpretation due to concerns regarding multiple testing, allele-frequency variation, and limited statistical power.

## Introduction

Gestational diabetes mellitus (GDM) is a prevalent pregnancy complication characterized by abnormal glucose metabolism in women who had normal glucose levels prior to pregnancy or who may have undiagnosed glucose intolerance, typically resolving after childbirth [1]. GDM poses significant health risks to both mothers and their offspring. Poor glycemic control can lead to maternal complications, including miscarriage, gestational hypertension, and an increased risk of type 2 diabetes, while elevated maternal glucose levels are associated with adverse neonatal outcomes such as macrosomia, respiratory distress, and hypoglycemia [2]. The global prevalence of gestational diabetes is rapidly increasing. In Europe, the overall prevalence is estimated at 10.9%, with the highest rates in Eastern Europe (31.5%) and the lowest in Northern Europe (8.9%), while Poland reports a prevalence of 6.2%. In North America and the Caribbean, the prevalence is 7.1%, in South and Central America it is 10.4%, and in Asia it varies widely from 1.2% to 49.5% [3]. In addition to established risk factors such as advanced maternal age and obesity, genetic susceptibility plays a significant role in the pathogenesis of GDM [4, 5].

Peroxisome proliferator-activated receptor (PPAR), a ligand-activated nuclear receptor, is integral to adipocyte differentiation, lipid metabolism, and insulin sensitivity [6]. Variants in the *PPARγ* gene may thus impact glucose homeostasis and the risk of developing GDM. Among these, the Pro12Ala polymorphism (a C→G missense mutation in exon 2 resulting in a proline-to-alanine substitution) is the most extensively studied [7, 8]. Functional studies indicate that the G (Ala) allele may enhance insulin sensitivity and reduce the risk of diabetes [9].

Nevertheless, evidence linking the Pro12Ala (Pro [C]→Ala [G]) polymorphism to GDM risk remains inconsistent across various studies and populations. A previous meta-analysis published in 2016 [8] suggested a potential protective effect of the Ala (G) allele, but was limited by a small sample size, incomplete subgroup analyses, and a lack of recent data. Since then, numerous studies with larger cohorts and diverse ethnic representation have emerged, yet the results continue to be contradictory. To address these discrepancies, we conducted an updated and comprehensive meta-analysis to reassess the association between the peroxisome proliferator-activated receptor *γ2* [*PPARγ2*] Pro12Ala (Pro [C]→Ala [G]) polymorphism and GDM risk, with the goal of better characterizing its genetic implications and providing robust evidence for future research.

## Materials and methods

### Subjects

The diagnostic criteria for the case group conformed to the standards for GDM set forth by the International Association of Diabetes and Pregnancy Study Groups (IADPSG) [10], using a 75 g oral glucose tolerance test (OGTT). Venous blood samples were collected at three time points: fasting (prior to glucose intake), 1 h post-ingestion, and 2 h post-ingestion. A diagnosis of GDM was made if any of the following plasma glucose concentrations met or exceeded the specified thresholds: (1) fasting glucose ≥5.1 mmol/L; (2) 1-h post-load glucose ≥10.0 mmol/L; (3) 2-h post-load glucose ≥8.5 mmol/L.

### Inclusion and exclusion criteria

Inclusion criteria were as follows: (1) studies reporting the association between *PPARγ2* and GDM risk, (2) case-control or cohort studies, (3) studies involving subjects who met the diagnostic criteria for GDM, and (4) studies that provided odds ratios (OR) and corresponding 95% confidence intervals (CI) based on the number of cases and genotyping methods used in both case and control groups. Exclusion criteria included: (1) non-case-control studies, and (2) duplicate publications or studies lacking necessary data. To prevent potential double counting, when multiple articles originated from the same research group or appeared to use overlapping cohorts, only the study with the largest or most complete dataset was included.

### Database

The following databases were utilized: (1) PubMed/Medline, (2) Web of Science, (3) Cochrane Library, and (4) Embase.

### Search strategy

A comprehensive literature search was conducted across PubMed/Medline, Web of Science, Embase, and the Cochrane Library from inception to August 2025, using combinations of Medical Subject Headings (MeSH) and free-text terms related to GSM and *PPARγ2* polymorphisms. The following search terms and their synonyms were employed: (“gestational diabetes mellitus” OR “GDM”) AND (“peroxisome proliferator-activated receptor gamma” OR “PPARG” OR “PPARγ” OR “PPARG2”) AND (“Pro12Ala” OR “rs1801282” OR “Proline12Alanine” OR “polymorphism” OR “variant” OR “mutation”). Boolean operators (AND, OR) were applied to appropriately combine terms. Filters were set to include only studies in English, involving human subjects, and classified as case-control or cohort studies. Reference lists of relevant reviews and meta-analyses were also manually screened to identify additional eligible studies.

### Study screening and data extraction

At least two investigators independently conducted the study screening. Initially, irrelevant studies were excluded based on the title and abstract, after which the full text of the remaining studies was obtained for further evaluation. The following data were extracted: basic study information (author, year of publication, study site, etc.), sample size, study population, genotype, etc. Any disagreements were resolved through consultation with a third investigator. Moreover, potential overlapping populations were meticulously examined across publications from the same institutions or author groups. Sensitivity analyses were carried out by sequentially excluding these studies to ensure that the pooled results were not influenced by overlapping cohorts.

### Quality evaluation

The quality of the included studies was assessed using the Newcastle–Ottawa Scale (NOS). This tool evaluates studies across three domains: (1) selection of study groups, (2) comparability of groups, and (3) ascertainment of outcomes. Each study could receive a maximum of nine stars, with higher scores indicating superior methodological quality. Studies with NOS scores of ≥7 were classified as high-quality studies. Two independent reviewers conducted the assessment, and any discrepancies were resolved through discussion.

### Statistical analysis

Meta-analyses were performed using RevMan 5.3, Stata 18.0, and R software (metafor and meta packages). Effect sizes were initially calculated as log ORs with corresponding standard errors, which were then exponentiated and expressed as ORs with 95% CIs for interpretation. Given the design of the genetic association studies and the limited number of included studies, we adhered to current methodological recommendations and employed a random-effects model with the Hartung–Knapp adjustment, irrespective of the level of heterogeneity. Prediction intervals (PIs) were estimated to determine the expected range of true effects in future studies. Statistical heterogeneity was assessed using the *Q*-test, *I*^2^, and τ^2^ statistics. Publication bias was evaluated using funnel plots, Begg’s test, Egger’s regression, and trim-and-fill analysis. For the model with non-significant Egger tests (i.e., the recessive model), no studies were imputed (*k*_0_ ═ 0), and the original pooled estimate was maintained. Sensitivity analyses were performed by sequentially excluding individual studies. Meta-regression was not feasible due to the number of studies per subgroup being <10, which would yield unstable estimates. As prespecified alternatives, we conducted influence diagnostics using Baujat plots and a leave-one-out (LOO) analysis. Study influence was quantified as the absolute change in the pooled OR following the sequential omission of each study. Subgroup analyses were stratified by ethnicity, study quality, and sample size.

## Results

### Search results and study selection

**Figure 1.**
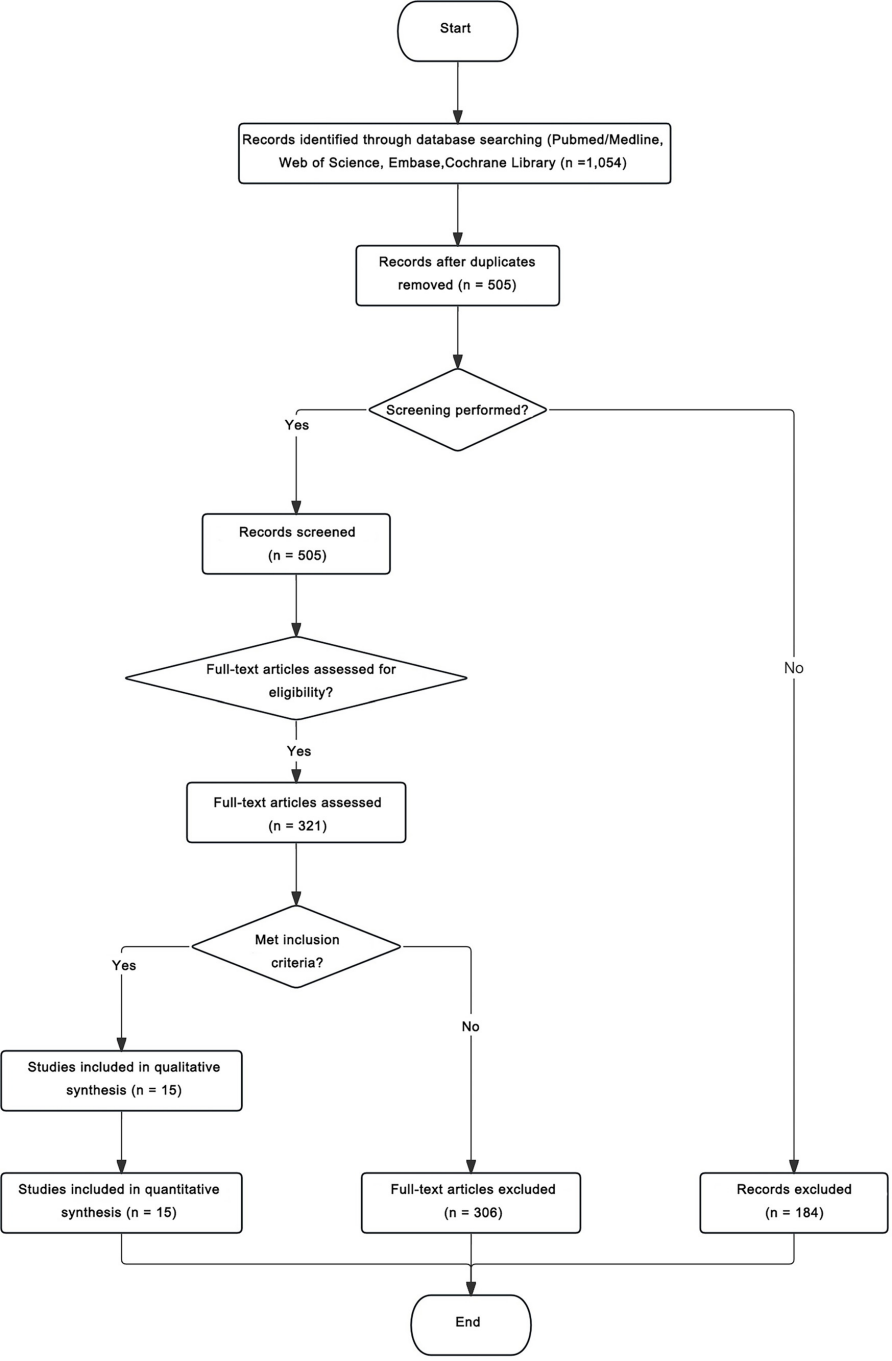
PRISMA flow diagram of study selection process.

A total of 1,054 records were retrieved from PubMed/Medline, Embase, Web of Science, and the Cochrane Library. After removing 549 duplicates, 505 records remained for screening. Based on titles and abstracts, 184 irrelevant studies were excluded, and 321 full-text articles were assessed for eligibility. Of these, 306 articles were excluded due to non-case-control design, insufficient data, animal or *in vitro* studies, conference abstracts, reviews, or overlapping cohorts. Ultimately, 15 studies met the inclusion criteria and were incorporated into both qualitative and quantitative synthesis (Figure 1).

### Basic characteristics of included studies

The included studies comprised a total of 12,760 subjects, including 4085 in the case group and 8675 in the control group [7, 11–24]. All studies were observational case-control investigations, with five conducted in East Asia, seven in Europe, one in the Middle East, one spanning the Middle East and Europe, and one in the Northeast. In addition to demographic characteristics, the diagnostic criteria for GDM and the timing of testing (gestational weeks) were extracted and are presented in Table 1 to standardize phenotyping across studies.

**Table 1:** Fundamental characteristics of included studies

**First author (year)**	**Race**	**Sample size**		**Genotype CC/CG/GG**		**Allele C/G**		**Study design**	**Diagnostic criteria**	**Timing of diagnosis (GA, weeks)**
		**Case group**	**Control group**	**Case group**	**Control group**	**Case group**	**Control group**			
Lauenborg et al., 2009 Cheng et al., 2010	Europe East Asia	265 55	2383 173	201/60/4 52/3/0	1790/542/51 157/16/0	462/68 107/3	4122/644 330/16	Case-control Case-control	WHO WHO	At 24 to 28 weeks At 24 to 28 weeks
Chon et al., 2013	East Asia	94	41	89/5/0	34/7/0	183/5	75/7	Case-control	IWC	At 24 to 28 weeks
Heude et al., 2011	Europe	109	1587	92/17/0	1265/305/17	201/17	2835/339	Case-control	IWC	Undefined
Pappa et al., 2011	Europe	148	107	143/5/0	100/7/0	291/5	207/7	Case-control	ADA	Undefined
Shaat et al., 2004	Middle East/Europe	500	550	377/120/3	423/120/7	874/126	966/134	Case-control	Other criteria	Undefined
Shaat et al., 2007	Europe	637	1232	468/158/11	918/298/16	1094/180	2134/330	Case-control	EASD	At 24 to 28 weeks
Tok et al., 2006	Middle East	62	100	50/12/0	84/16/0	112/12	184/16	Case-control	NDDG	At 24 to 28 weeks
Cho et al., 2009	East Asia	865	632	793/71/1	567/63/2	1657/73	1197/67	Case-control	IWC	At 24 to 28 weeks
Yan et al., 2020	East Asia	156	180	144/12/0	153/24/3	300/12	330/30	Case-control	Other criteria	Undefined
Kuzmicki et al., 2013	Europe	20	20	18/2/0	17/3/0	38/2	37/3	Case-control	PDA	Undefined
Rosta et al., 2017	Europe	217	670	168/46/3	507/152/11	382/55	1166/174	Case-control	IADPSG	Undefined
Franzago et al., 2018 Shen et al., 2020 Bhushan et al., 2024	Europe East Asia South Asia	104 753 100	124 676 200	79/25/0 676/77/0 75/25/0	101/23/0 589/81/2 171/25/4	183/25 1429/77 175/25	225/231 259/85 367/33	Case-control Case-control Case-control	IADPSG IADPSG WHO	At 24 to 28 weeks At 24 to 28 weeks At 24 to 28 weeks

Abbreviations: WHO: World Health Organization; IWC: International Workshop-Conference; ADA: American Diabetes Association; EASD: European Association for the Study of Diabetes; NDDG: U.S. National Diabetes Data Group; PDA: Polish Diabetes Association; IADPSG: International Association of Diabetes in Pregnancy Study Groups.

### Quality assessment of included studies

The methodological quality of the included case-control studies was evaluated using the NOS. In the selection domain, three criteria were assessed: (1) Representativeness of cases: whether the cases accurately represented individuals in the target population who developed the disease of interest; (2) Selection of controls: whether the controls were appropriately chosen (e.g., community-based or hospital-based); (3) Definition and representativeness of controls: whether the controls adequately represented the non-diseased population from which the cases originated. In the comparability domain, two criteria were considered: (4) Comparability of cases and controls with respect to key confounding factors (such as age and sex); (5) Comparability regarding other potential confounders beyond the primary exposure of interest. In the exposure assessment domain, three criteria were evaluated: (6) Ascertainment of exposure among cases; (7) Ascertainment of exposure among controls; and (8) Accuracy and reliability of exposure measurement, including additional factors related to measurement precision, consistency, and methodological rigor in capturing true exposure levels. Most studies received high scores for participant selection and outcome ascertainment. The comparability between groups, a core NOS criterion, was also well addressed in the majority of studies. Overall, the included studies demonstrated satisfactory methodological quality, meeting the expected standards for inclusion in this meta-analysis (Table 2).

**Table 2:** Assessment of quality in included studies

**Studies**	**1**	**2**	**3**	**4**	**5**	**6**	**7**	**8**	**9**	**Score**	**Overall of quality**	**Reasons**
Lauenborg et al., 2009	1	1	0	1	0	1	0	1	1	6	Moderate	Insufficient matching of exposure background; incomplete quality control records; inadequate control of confounding factors
Cheng et al., 2010	1	1	1	0	1	1	0	1	1	7	High	Age difference not statistically tested; key GDM-related features unreported; potential confounding bias due to lack of adjustment
Chon et al., 2013	1	1	1	0	1	1	0	0	1	6	Moderate	Limited comparability of key traits; unadjusted confounders; lack of blinding in genotyping may introduce bias
Heude et al., 2011	1	1	1	1	1	1	0	1	1	8	High	Unadjusted inter-center threshold differences and incomplete center effect control
Pappa et al., 2011	1	1	1	0	1	1	0	0	1	6	Moderate	Limited comparability of key features, unadjusted core confounders, and lack of blinding in exposure assessment
Shaat et al., 2004	1	1	1	1	1	1	0	1	1	8	High	Incomplete confounding control
Shaat et al., 2007	1	1	1	1	1	1	1	1	0	8	High	HWE P-values not reported; insufficient detail on population representativeness
Tok et al., 2006	1	1	1	1	0	1	1	1	1	8	High	Incomplete quality control measures, limiting confirmation of genotyping reliability
Cho et al., 2009	1	1	1	1	1	1	0	1	1	7	High	Possible prior GDM history in controls not excluded; confounding control incomplete
Yan et al., 2020	1	1	1	0	1	1	1	1	1	7	High	Age data missing; incomplete baseline traits and unverified group comparability
Kuzmicki et al., 2013	1	1	1	0	1	1	1	1	1	8	High	Lack of QC details limits assay reliability verification
Rosta et al., 2017	1	1	1	1	1	1	1	1	0	8	High	No comprehensive correction provided for multiple testing of 77 SNPs
Franzago et al., 2018	1	1	1	1	0	0	1	1	1	6	Moderate	Age/BMI imbalance unaddressed; genotyping QC data insufficient
Shen et al., 2020	1	1	1	1	1	1	0	1	1	8	High	Hospital source differences unadjusted; potential assay or procedural variations may introduce residual confounding
Bhushan et al., 2024	1	1	1	1	1	1	0	1	1	8	High	No multivariable adjustment reported; confounding control uncertain

Abbreviations: GDM: Gestational diabetes mellitus; BMI: Body mass index; SNP: Single nucleotide polymorphism; HWE: Hardy–Weinberg equilibrium.

### Hardy–Weinberg equilibrium test

The results of the Hardy–Weinberg equilibrium test indicated that the control groups in 14 studies conformed to Hardy–Weinberg equilibrium (*P* > 0.05). However, in the study by Bhushan et al. (2024), which involved a South Asian population, the control group data did not conform to Hardy–Weinberg equilibrium (*P* < 0.05, Table 3).

**Table 3:** Hardy–Weinberg equilibrium test: Expected genotype counts under HWE

**Studies**	**Allele C frequency (P)**	**Allele G frequency (P)**	**Genotype CC/CG/GG frequency (P)**	**χ^2^**	***P***	**HWE conformity**
Lauenborg et al., 2009	0.865	0.135	1784.6/555.0/43.4	2.03	0.154	Yes
Cheng et al., 2010	0.954	0.046	157.5/15.0/0.4	0.05	0.829	Yes
Chon et al., 2013	0.915	0.085	34.3/6.3/0.3	0.19	0.665	Yes
Heude et al., 2011	0.893	0.107	1265.6/303.9/17.5	0.02	0.888	Yes
Pappa et al., 2011	0.967	0.033	100.1/6.8/0.1	0.01	0.907	Yes
Shaat et al., 2004	0.878	0.122	423.9/120.2/5.9	0.28	0.599	Yes
Shaat et al., 2007	0.866	0.134	924.6/295.9/11.5	2.66	0.103	Yes
Tok et al., 2006	0.920	0.080	84.6/14.7/0.6	0.38	0.536	Yes
Cho et al., 2009	0.947	0.053	567.0/63.1/1.8	0.02	0.900	Yes
Yan et al., 2020	0.917	0.083	151.4/27.3/1.2	3.20	0.074	Yes
Kuzmicki et al., 2013	0.925	0.075	17.1/2.8/0.1	0.04	0.835	Yes
Rosta et al., 2017	0.870	0.130	507.7/151.8/11.1	0.00	0.982	Yes
Franzago et al., 2018	0.907	0.093	102.0/21.4/0.6	0.43	0.512	Yes
Shen et al., 2020	0.931	0.069	586.0/86.1/3.2	1.54	0.215	Yes
Bhushan et al., 2024	0.918	0.083	168.4/30.5/1.4	4.27	0.039	No

Abbreviation: HWE: Hardy–Weinberg equilibrium.

### Allelic model (Ala [G] vs Pro [C])

**Figure 2.**
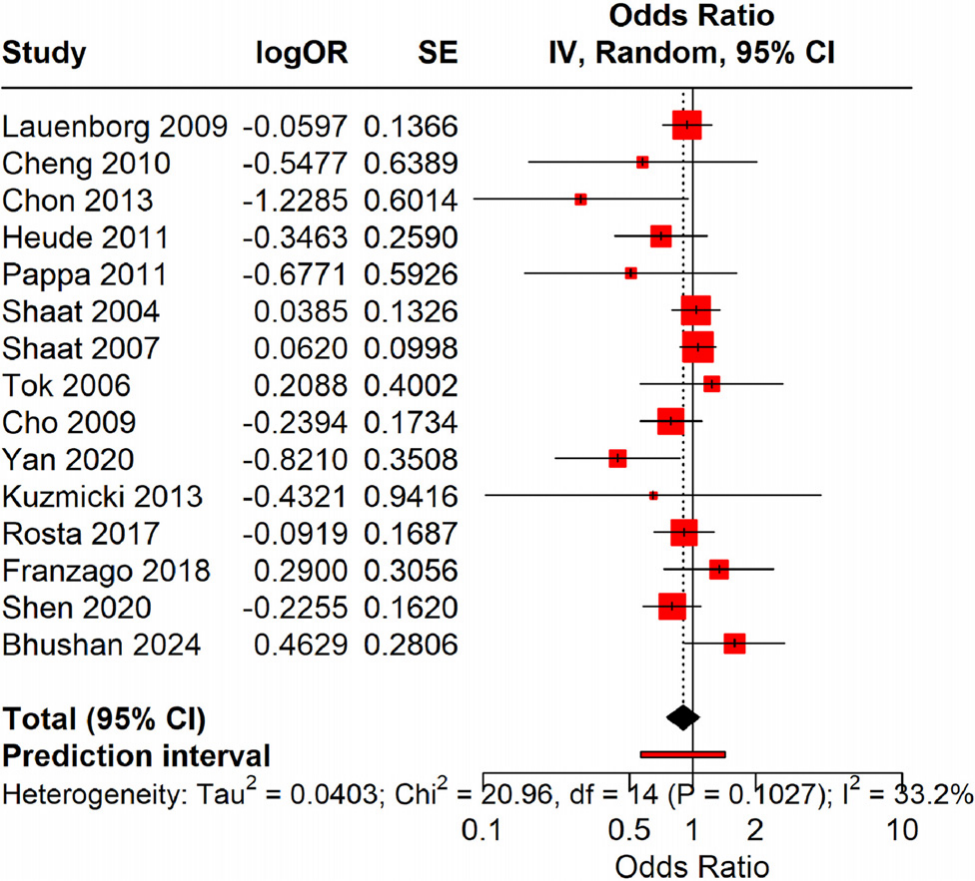
**Forest plot illustrating the allelic model (Ala [G] vs Pro [C]).** Pooled odds ratios (OR) and 95% confidence intervals (CIs) were calculated to assess the association between the Ala (G) allele and the risk of gestational diabetes mellitus (GDM), utilizing the Hartung–Knapp random-effects model. The summary odds ratio was 0.90 (95% CI: 0.75–1.08, *P* ═ 0.26), accompanied by a 95% PI of 0.63–1.29 and a modest degree of heterogeneity (*I*^2^ ═ 33.2%, τ^2^ ═ 0.0403), indicating no significant association.

A meta-analysis utilizing the Hartung–Knapp random-effects model revealed no significant association between the Ala allele and the risk of GDM. The pooled OR was 0.90, with a 95% CI of 0.75–1.08 (*P* ═ 0.26). The 95% PI was 0.63–1.29, suggesting that future studies are also unlikely to demonstrate a strong association. Heterogeneity was modest (*I*^2^ = 33.2%, τ^2^ ═ 0.0403). Overall, these results indicate no significant association between the Ala allele and GDM risk (Figure 2).

### Dominant model (Ala carriers [CG+GG] vs Pro homozygotes [CC])

**Figure 3.**
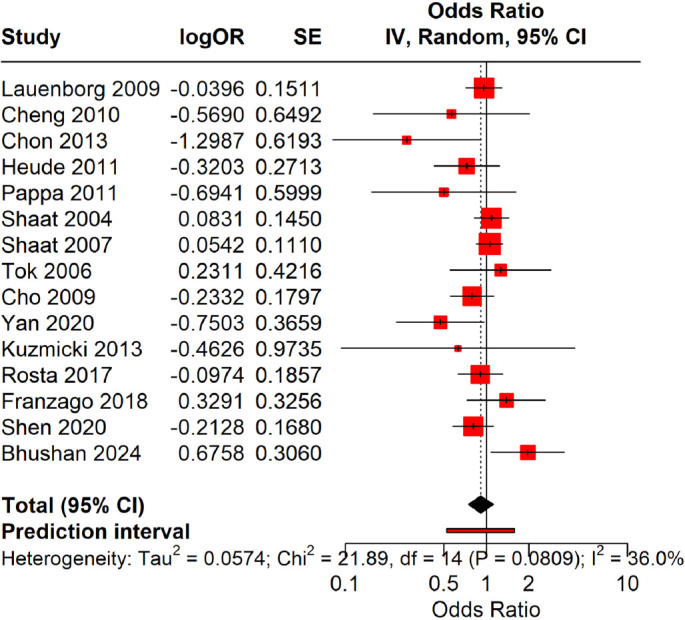
**Forest plot for the dominant model (combined homozygous and heterozygous carriers of Ala [CG+GG] versus homozygous Pro [CC]).** This figure presents study-specific and pooled OR with 95% CI for gestational diabetes mellitus (GDM) risk among Ala allele carriers (CG+GG) in comparison to Pro homozygotes (CC). Estimates were derived using the Hartung–Knapp random-effects model. The pooled OR was 0.92 (95% CI: 0.74–1.13; *P* ═ 0.42), with a 95% PI of 0.61–1.36 and moderate heterogeneity (*I*^2^ ═ 36%). These results indicate no significant association between the *PPARγ2* Pro12Ala variant and GDM risk under the dominant model.

Using the Hartung–Knapp random-effects model, carriers of the Ala allele (CG+GG) did not exhibit a significantly different risk of GDM compared to Pro homozygotes. The pooled OR was 0.92 (95% CI: 0.74–1.13), with a 95% PI of 0.61–1.36 (*P* ═ 0.42, *I*^2^ ═ 36%). These findings suggest no association between the *PPARγ2* Pro12Ala variant and GDM under the dominant model (Figure 3).

### Recessive model (Ala homozygotes [GG] vs Pro carriers [CG+CC])

**Figure 4.**
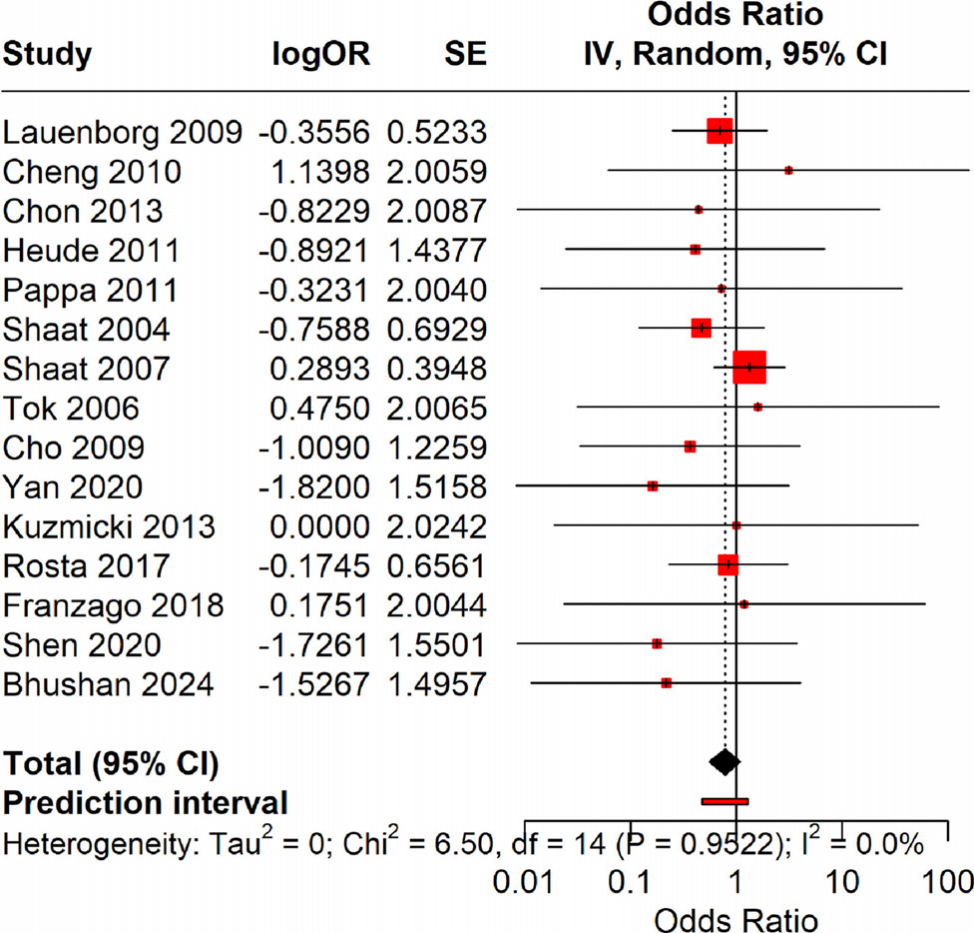
**Forest plot for the recessive model (GG vs CG+CC).** Utilizing the Hartung–Knapp random-effects model, no significant association was identified under the recessive model. The pooled odds ratio (OR) was 0.82 (95% confidence interval [CI]: 0.54–1.25; *P* ═ 0.33), with a 95% PI of 0.37–1.81 and *I*^2^ ═ 0%. These results indicate that homozygosity for the Ala (G) allele does not significantly alter the risk of gestational diabetes mellitus (GDM).

The Hartung–Knapp random-effects model showed no significant association under the recessive model. The pooled OR was 0.82 (95% CI: 0.54–1.25), with a 95% PI of 0.37–1.81 (*P* ═ 0.33, *I*^2^ ═ 0%). These findings indicate that homozygosity for the Ala (G) allele does not significantly alter the risk of GDM (Figure 4).

### Publication bias

**Figure 5.**
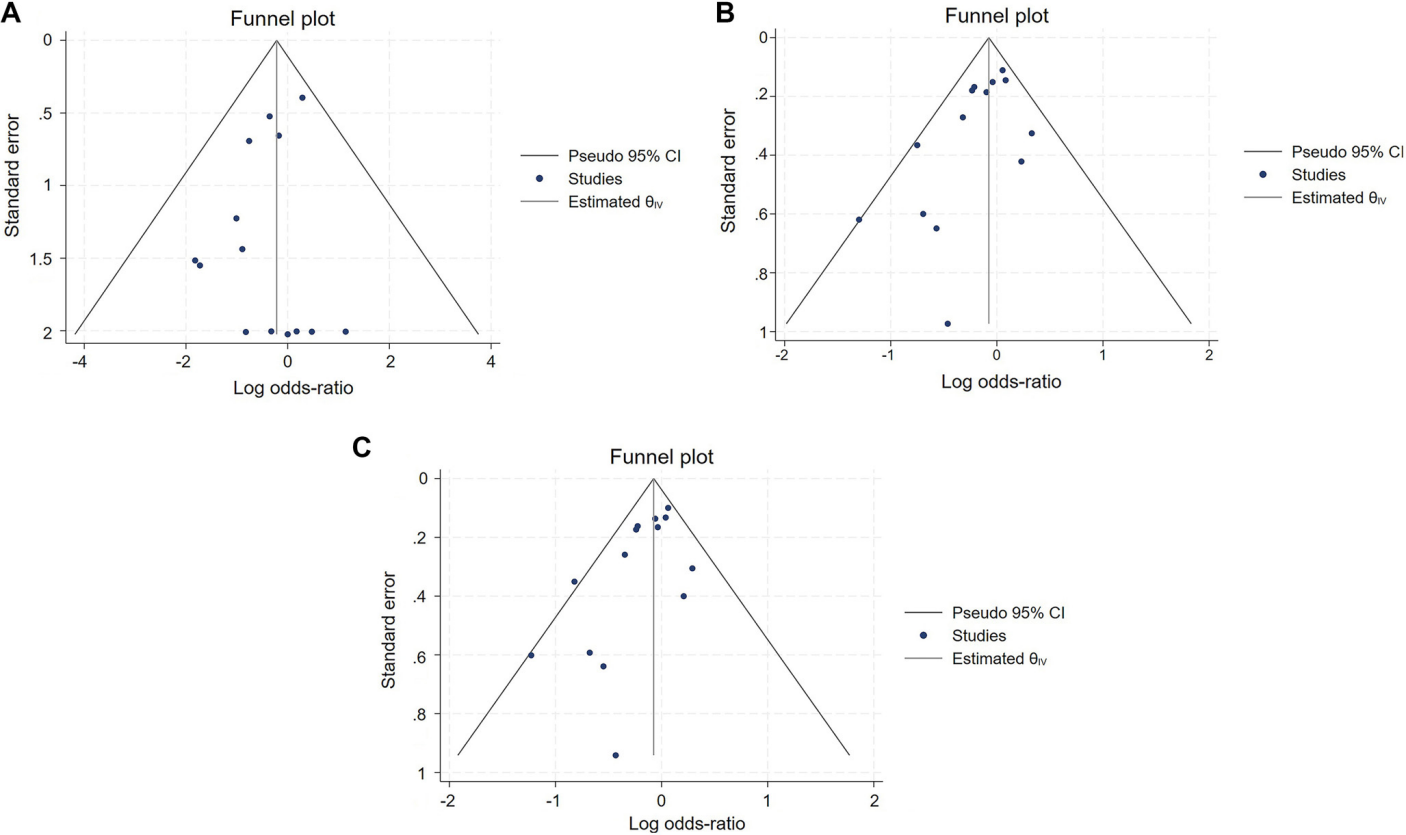
**Funnel plots illustrating the assessment of publication bias across studies.** (A) Funnel plot for the allelic model (Ala [G] vs Pro [C]); (B) Funnel plot for the dominant model (combined homozygous and heterozygous carriers, Ala [CG+GG] vs Pro homozygotes [CC]); (C) Funnel plot for the recessive model (GG vs CG+CC).

To assess potential publication bias, funnel plots and Egger’s test were performed for each genetic model (Figure 5). In the allelic model (Ala [G] vs Pro [C]), most scatter points were concentrated on the left side of the funnel plot, suggesting possible publication bias. Egger’s test confirmed this with *Z* ═ −2.41, *P* ═ 0.016, indicating a small-sample effect and the presence of publication bias. In the dominant model (Ala carriers [CG+GG] vs Pro homozygotes [CC]), the majority of points were similarly clustered on the left side of the funnel plot, indicating evident publication bias. Egger’s test yielded *Z* ═ −2.25, *P* ═ 0.0246, confirming the presence of publication bias. In contrast, the recessive model (Ala homozygotes [GG] vs Pro carriers [CG+CC]) exhibited a symmetrical distribution of scatter points, suggesting a lower likelihood of publication bias. The Egger’s test result (*Z* ═ −0.91, *P* ═ 0.3611) indicated no significant publication bias.

To further evaluate publication bias, Begg’s rank correlation test was conducted, which did not detect significant publication bias in any model (allelic: *P* ═ 0.15; dominant: *P* ═ 0.15; recessive: *P* ═ 0.66), although point estimates suggested a weak negative correlation. Trim-and-fill procedures for the allelic and dominant models yielded similar pooled estimates, indicating robustness of results (Figure S1). Trim-and-fill analysis imputed no missing studies (*k*_0_ ═ 0), indicating no evidence of publication bias under the recessive model. Collectively, while Egger’s test suggested small-study effects in the allelic and dominant models, the absence of significant findings in Begg’s test and the stability of trim-and-fill estimates support the overall conclusion of no material publication bias.

### Sensitivity and influence analyses

Sensitivity analyses were conducted by sequentially excluding each study included in the meta-analysis. In the allelic model (Ala [G] vs Pro [C]), the *I*^2^ values consistently remained below 50% upon the exclusion of any single study, indicating low heterogeneity. The pooled ORs were consistently less than 1, with the lowest heterogeneity (*I*^2^ ═ 8%) observed after removing Yan et al. (2020). These results suggest that the findings of the meta-analysis are robust. In the dominant model (Ala carriers [CG+GG] vs Pro homozygotes [CC]), sensitivity analysis also demonstrated *I*^2^ < 50% across all iterations, confirming a low level of heterogeneity. The combined ORs remained below 1, with minimal heterogeneity (*I*^2^ ═ 1%) when Chon et al. (2013) was excluded. This further substantiates the stability of the meta-analytic findings. In the recessive model (Ala homozygotes [GG] vs Pro carriers [CG+CC]), *I*^2^ consistently equaled 0%, with pooled ORs < 1 throughout the analysis, indicating that the results of the meta-analysis were highly stable and reliable.

Given that the number of studies per subgroup was fewer than ten, meta-regression was not feasible. As prespecified alternatives, we conducted influence diagnostics (Baujat plot and delta-influence analysis) and LOO sensitivity analysis. For the allelic model, the Baujat plot identified Yan et al. (2020) as the primary contributor to heterogeneity and the most influential study on the pooled estimate, followed by Chon et al. (2013) and Bhushan et al. (2024). However, the magnitude of this influence was small (all Δ-OR ≤ 0.03). The LOO analysis demonstrated that removal of any single study yielded stable estimates (pooled OR range 0.88–0.94), indicating the robustness of the results (Figure S2). The dominant and recessive models exhibited similar stability, with no single study materially altering the pooled effect or heterogeneity (Figures S3 and S4).

### Subgroup analysis

Subgroup analyses were performed based on ethnicity, study quality, and sample size (Table 4). Regarding ethnicity, in both the allelic (Ala [G] vs Pro [C]) and dominant (Ala carriers [CG+GG] vs Pro homozygotes [CC]) models, heterogeneity was low (*I*^2^ < 50%), with only the East Asian subgroup exhibiting a suggestive protective trend of the Ala (G) allele against GDM, while European and Middle Eastern groups did not show this trend. Between-group differences were significant (*P* < 0.05). In the recessive model (Ala homozygotes [GG] vs Pro carriers [CG+CC]), heterogeneity was negligible (*I*^2^ ═ 0%) with no significant subgroup differences.

**Table 4:** Subgroup analysis

**Grouping methods**	**Genetic model**	**Groups**	**Number of studies**	***I*^2^ (%)**				
					**Log OR**	**OR**	**95% CI**	***P***
Subgroup by ethnicity	G vs C	East Asia	5	19.98	--0.34	0.71	--0.55, --0.12	0.001
		Europe	8	0.00	0.00	1.00	--0.12, 0.12	0.98
		Middle East	2	24.84	--0.12	0.89	--0.68, 0.45	0.68
		Overall	15	25.13	--0.08	0.92	--0.18, 0.02	0.12
			Test of group difference: *Q* ═ 7.42, *P* ═ 0.02				
	CG+GG vs CC	East Asia	5	14.34	--0.32	0.73	--0.54, --0.10	0.001
		Europe	8	0.00	0.00	1.00	--0.13, 0.14	0.95
		Middle East	2	13.10	--0.09	0.91	--0.68, 0.50	0.77
		Overall	15	18.63	--0.08	0.92	--0.19, 0.03	0.15
			Test of group difference: *Q* ═ 6.25, *P* ═ 0.04				
	GG vs CG+CC	East Asia	5	0.00	--1.15	0.32	--2.47, 0.17	0.09
		Europe	8	0.00	--0.13	0.88	--0.62, 0.36	0.60
		Middle East	2	0.00	--0.40	0.67	--2.79, 1.99	0.74
		Overall	15	0.00	--0.27	0.76	--0.72, 0.17	0.23
			Test of group difference: *Q* ═ 2.02, *P* ═ 0.36				
Subgroup by quality of research	G vs C	High	10	20.04	--0.08	0.92	--0.19, 0.03	0.18
		Moderate	4	51.22	--0.08	0.92	--0.31, 0.16	0.52
		Overall	14	25.31	--0.08	0.92	--0.18, 0.02	0.13
			Test of group difference: *Q* ═ 0.00, *P* ═ 0.99				
	CG+GG vs CC	High	10	5.24	--0.08	0.92	--0.20, 0.04	0.19
		Moderate	4	54.28	--0.07	0.93	--0.32, 0.19	0.61
		Overall	14	19.07	--0.08	0.92	--0.19, 0.03	0.16
			Test of group difference: *Q* ═ 0.01, *P* ═ 0.92				
	GG vs CG+CC	High	10	0.00	--0.26	0.77	--0.77, 0.26	0.33
		Moderate	4	0.00	--0.35	0.71	--1.28, 0.59	0.46
		Overall	14	0.00	--0.28	0.76	--0.73, 0.17	0.23
			Test of group difference: *Q* ═ 0.03, *P* ═ 0.86				
Subgroup by sample size	G vs C	Large	3	13.79	--0.11	0.90	--0.28, 0.06	0.21
		Medium	7	38.48	--0.05	0.95	--0.18, 0.08	0.49
		Small	4	28.02	--0.31	0.73	--0.86, 0.23	0.26
		Overall	14	25.31	--0.08	0.92	--0.18, 0.02	0.13
			Test of group difference: *Q* ═ 1.09, *P* ═ 0.58				
	CG+GG vs CC	Large	3	22.85	--0.09	0.91	--0.28, 0.09	0.31
		Medium	7	26.10	--0.05	0.95	--0.19, 0.09	0.47
		Small	4	31.38	--0.34	0.71	--0.90, 0.23	0.24
		Overall	14	19.07	--0.08	0.92	--0.19, 0.03	0.16
			Test of group difference: *Q* ═ 0.95, *P* ═ 0.62				
					**Log OR**	**OR**	**95% CI**	***P***
	GG vs CG+CC	Large	3	0.00	--0.96	0.38	--2.06, 0.13	0.08
		Medium	7	0.00	--0.14	0.87	--0.66, 0.37	0.58
		Small	4	0.00	0.18	1.20	--1.79, 2.15	0.86
		Overall	14	0.00	--0.28	0.76	--0.73, 0.17	0.23
			Test of group difference: *Q* ═ 1.98, *P* ═ 0.37				

In terms of study quality, both high- and moderate-quality studies exhibited low to moderate heterogeneity and no significant associations across all genetic models. Between-group differences were non-significant (*P* > 0.05). Regarding sample size, all subgroups demonstrated low or no heterogeneity and no significant associations in any model, with non-significant between-group differences (*P* > 0.05). Overall, ethnicity appeared to be a key effect modifier, while study quality and sample size had minimal influence on the pooled results. Following trim-and-fill adjustment for potential publication bias, the East Asian subgroup maintained a similar protective direction and magnitude, with no substantial change in statistical significance, further suggesting the robustness of the observed trend.

## Discussion

This meta-analysis synthesizes data from 15 case-control studies to comprehensively investigate the association between the *PPARγ2* gene Pro12Ala (Pro [C]→Ala [G]) polymorphism and the risk of GDM. *PPARγ2*, a member of the nuclear hormone receptor superfamily, is crucial for lipid metabolism, glucose homeostasis, and insulin sensitivity. The Pro12Ala missense mutation, a variant exclusive to the *PPARγ2* isoform, has been widely associated with a decreased risk of type 2 diabetes. This mutation arises from a C→G substitution at codon 12 in exon 2, resulting in the substitution of proline (Pro) with alanine (Ala) and representing one of the most common variants of the *PPARγ* gene [25]. Insulin phosphorylation can enhance ligand-dependent activation of the N-terminal domain of *PPARγ*, indicating a close relationship between insulin signaling and *PPARγ* functionality [26]. The presence of the Pro12Ala polymorphism may modify this interaction, thus affecting cellular insulin responsiveness and lipid metabolism. Nonetheless, the role of this genetic variant in the specific physiological context of GDM remains debated. The current pooled analysis revealed no significant association between the Pro12Ala polymorphism and GDM risk across any of the three genetic models. Subgroup analyses indicated potential ethnic differences, but these findings should be interpreted cautiously due to multiple testing, allele-frequency variation, and limited statistical power in some comparisons.

Across all genetic models, the pooled ORs were near 1, with CIs crossing the null value, suggesting that Pro12Ala is unlikely to be a significant susceptibility locus for GDM. This “negative” finding may reflect the complex pathophysiology of GDM, characterized by insulin resistance and inadequate compensatory β-cell function. The mild insulin-sensitizing effect of the Ala (G) allele may not compensate for this physiological burden. Additionally, pooling studies from diverse genetic backgrounds may have obscured subgroup-specific effects.

Previous research has indicated potential ethnic differences in GDM prevalence [27]. In analyses stratified by ethnicity, the Ala (G) allele exhibited a suggestive protective trend in East Asian populations under allelic and dominant models, while no association was observed in European or Middle Eastern cohorts. Given the limited number of studies and multiple comparisons, this observation should be considered exploratory. This pattern may reflect gene–environment interactions, as lifestyle, adiposity, and dietary factors vary across ethnic groups. Variations in allele frequencies and linkage disequilibrium structures may also play a role.

Placing these findings in a broader academic context, the overall null association contrasts with earlier small-sample studies, underscoring the greater reliability of conclusions drawn from pooled data. The ethnicity-related trend noted here aligns with findings from studies on *PPARγ2* and type 2 diabetes but remains hypothesis-generating due to limited statistical power, particularly under the recessive model where the Ala/Ala genotype is rare.

Publication bias analyses employing Egger’s, Begg’s, and trim-and-fill methods suggested minor small-study effects without materially altering pooled estimates. Sensitivity and influence diagnostics confirmed the robustness of the results, and Hardy–Weinberg equilibrium testing validated the genetic integrity of the controls. Collectively, these findings reinforce the stability and methodological rigor of the meta-analysis, while acknowledging that undetected negative studies could render the overall “no association” conclusion conservative.

This study, however, has several limitations. First, as a meta-analysis reliant on aggregated literature data, individual-level data were unavailable, making it challenging to accurately adjust for pre-pregnancy body mass index (BMI), a significant confounding factor for GDM. Second, diagnostic criteria for GDM varied slightly across studies. Although most studies performed adequately according to quality assessments, such inconsistencies may still represent a potential source of heterogeneity. Third, the relatively limited number of studies in certain strata, particularly in the recessive model, and the multiple subgroup comparisons may increase the likelihood of type I error.

## Conclusion

In conclusion, this meta-analysis found no statistically significant association between the *PPARγ2* Pro12Ala (Pro [C]→Ala [G]) polymorphism and the overall risk of GDM across all genetic models. Compared to earlier meta-analyses, this study incorporated more recent data, applied genetics-appropriate quality assessments, and conducted detailed subgroup and sensitivity analyses, resulting in a more robust and reliable pooled estimate. A suggestive protective trend of the Ala (G) allele among East Asian populations was observed but remains inconclusive due to limited statistical power and multiple testing. Overall, the *PPARγ2* Pro12Ala polymorphism may have only a minor influence on GDM susceptibility, necessitating confirmation through large, multicenter studies utilizing standardized protocols and multivariate analyses.

## Supplemental data

Supplemental data are available at the following link: https://www.bjbms.org/ojs/index.php/bjbms/article/view/13079/4049.

## Data Availability

The extracted data are provided in the supplementary materials.
